# Uniportal Video‐Assisted Thoracoscopic Surgery for Intrathoracic Phrenic Nerve Schwannomas: A Report of 2 Cases and Literature Review

**DOI:** 10.1155/cris/2057048

**Published:** 2026-11-15

**Authors:** Junjun Ni, Minglei Yang, Rui Li, Yangbo Qiu

**Affiliations:** ^1^ Department of Thoracic Surgery Ningbo No. 2 Hospital Wenzhou Medical University Ningbo 315000 Zhejiang Province China wmu.edu.cn

**Keywords:** case report, nerve function preservation, phrenic nerve, phrenic nerve schwannoma, uniportal video-assisted thoracoscopic surgery

## Abstract

Phrenic nerve schwannoma (PNS) is an extremely rare benign peripheral nerve sheath tumor with atypical clinical manifestations, leading to frequent preoperative misdiagnosis. This study aimed to explore the clinical efficacy and surgical strategy of uniportal video‐assisted thoracoscopic surgery (uni‐VATS) for intrathoracic PNS. Two asymptomatic patients with intrathoracic PNS admitted to our hospital were enrolled, including one 55‐year‐old male with a left anterior mediastinal mass and one 22‐year‐old female with a right middle mediastinal mass. Preoperative contrast‐enhanced chest computed tomography (CT) and magnetic resonance imaging (MRI) demonstrated well‐circumscribed cystic‐solid mediastinal masses with heterogeneous enhancement. Both patients received uni‐VATS treatment: end‐to‐end phrenic nerve anastomosis was performed after tumor resection for the first case due to dense tumor‐nerve adhesion, while intracapsular enucleation was adopted for the second case with clear dissection planes. The operative time was 69 and 60 min, intraoperative blood loss was 10 mL in both cases, and postoperative hospital stay was 3 days for both patients without perioperative complications. Postoperative histopathology and immunohistochemistry confirmed benign schwannoma with positive S‐100 and SOX10 expression. During follow‐up, only mild asymptomatic left diaphragmatic elevation was observed in the first case, and no tumor recurrence or nerve dysfunction occurred in either case. Uni‐VATS is a safe and minimally invasive approach for localized intrathoracic PNS. Individualized surgical strategies based on tumor‐nerve anatomical characteristics can maximize phrenic nerve function preservation and achieve favorable long‐term prognosis.

## 1. Introduction

Phrenic nerve schwannoma (PNS) is a rare benign neoplasm originating from Schwann cells of the phrenic nerve sheath, first documented by Sarot IA in 1969 [1], and accounts for less than 2% of all peripheral nerve sheath tumors. This lesion can occur along any segment of the phrenic nerve pathway, with predilection for the C3–C5 cervical segment and the thoracic middle mediastinum [2]. Given the concealed anatomical location of the intrathoracic phrenic nerve and the nonspecific early clinical features of PNS, the majority of patients are diagnosed incidentally during routine health check‐ups; a minority of patients present with chest tightness, chest pain, intractable hiccups, or related symptoms secondary to phrenic nerve palsy or tumor compression of surrounding tissues, leading to frequent misdiagnosis as other neurogenic tumors, thymomas, or mediastinal cysts [3–5].

Surgical resection remains the radical therapeutic modality for PNS. Traditional thoracotomy has been progressively supplanted by thoracoscopic surgery due to its inherent drawbacks, including extensive surgical trauma, delayed postoperative recovery, and a relatively high complication rate [5, 6]. As an advanced iteration of thoracoscopic minimally invasive techniques, uniportal video‐assisted thoracoscopic surgery (uni‐VATS) has gained widespread clinical application in the management of diverse intrathoracic diseases in recent years, attributed to its merits of fewer incisions, mild postoperative pain, rapid rehabilitation, and excellent cosmetic results [7–10]. Nevertheless, owing to the extreme rarity of PNS cases, clinical reports focusing on uni‐VATS for this disorder remain scarce, and the key surgical techniques and individualized treatment paradigms warrant further summarization and refinement. This study retrospectively analyzed the clinical data of 2 cases with intrathoracic PNS successfully managed via uni‐VATS at our institution, accompanied by a comprehensive literature review, aiming to provide clinical reference for the diagnosis and treatment of this rare condition.

## 2. Case Presentation

### 2.1. Case 1

A 55‐year‐old male patient was admitted to our hospital in September 2024 with a chief complaint of “incidentally detected left anterior mediastinal mass for 5 years.” The patient was found to have a 12 mm nodule in the lingular segment of the left upper lobe on chest computed tomography (CT) during routine physical examination 5 years ago (Figure 1A) and failed to comply with regular follow‐up as recommended. Two weeks prior to admission, repeated plain chest CT at a local hospital revealed tumor enlargement to 25 mm × 21 mm, with close proximity to the pericardium. The patient was asymptomatic at admission, with no complaints of chest pain, chest tightness, dyspnea, cough, or expectoration. He had no significant past medical history and no familial history of malignant tumors. Physical examination revealed midline trachea, no enlargement of systemic superficial lymph nodes, clear bilateral breath sounds, and unremarkable cardiac and abdominal findings, with an Eastern Cooperative Oncology Group (ECOG) performance status score of 0.

**Figure 1:**
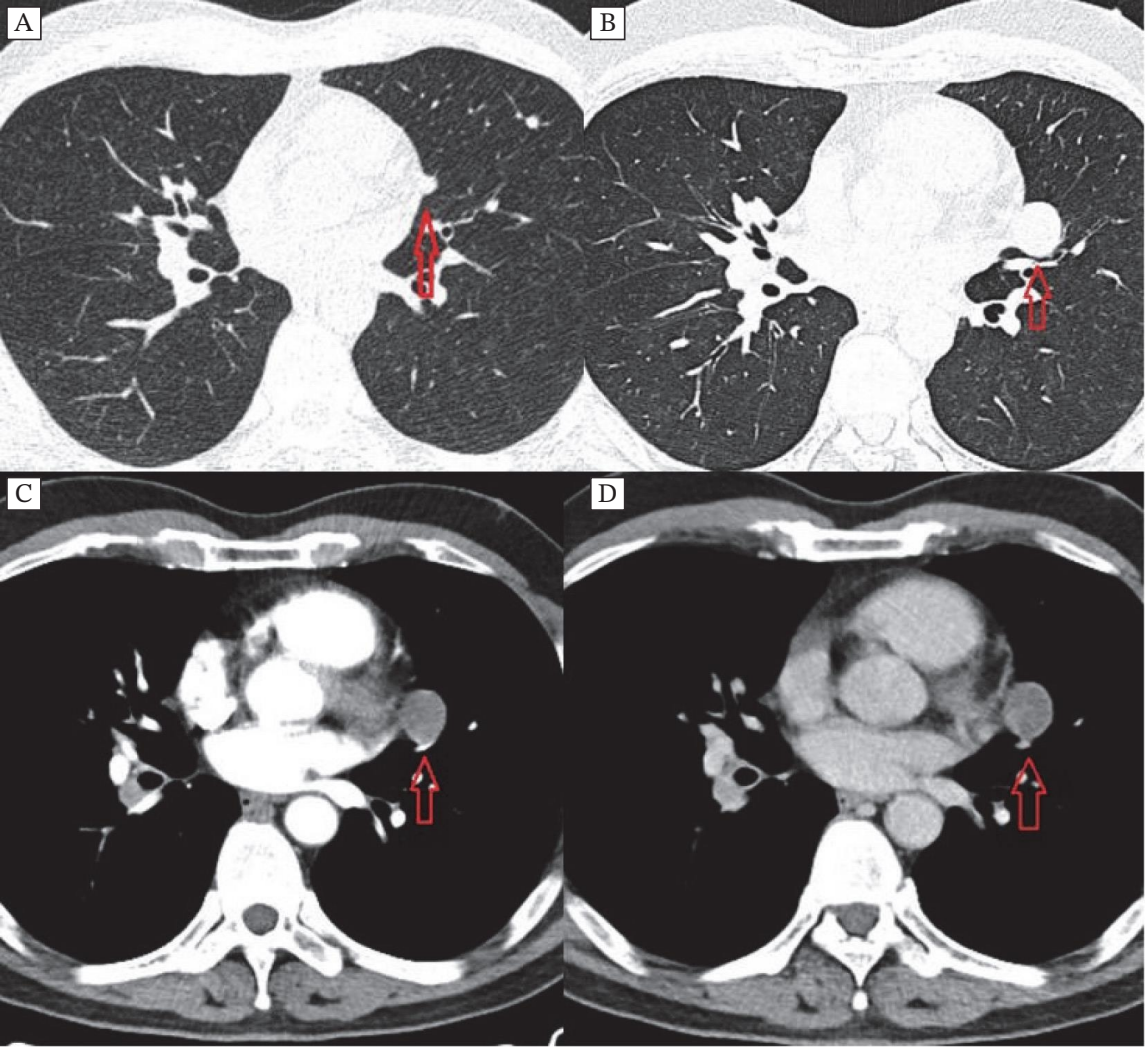
Chest CT findings of Case 1. (A) Preoperative noncontrast chest CT obtained 5 years before admission showing a small nodule in the left upper lobe lingular segment (arrow indicates the lesion); (B) admission plain chest CT showing enlarged mediastinal mass (arrow indicates the tumor); (C) arterial phase of contrast‐enhanced chest CT demonstrating mild heterogeneous enhancement of the lesion (arrow indicates the tumor); (D) venous phase of contrast‐enhanced chest CT showing continuous enhancement characteristics of the mass (arrow indicates the tumor).

After admission, contrast‐enhanced chest CT was performed, demonstrating a 23 mm × 19 mm solid nodule adjacent to the mediastinal surface of the left upper lobe lingular segment, with clear demarcation and mild heterogeneous enhancement. The triphasic CT values were ~27 HU, 51 HU, and 33 HU, respectively, suggestive of a mediastinal‐origin neoplasm (Figure 1B–D). Preoperative routine laboratory examinations, including blood routine, hepatic and renal function, and coagulation profile, revealed no obvious abnormalities, and CT‐guided fine‐needle aspiration biopsy was not performed.

The patient underwent uni‐VATS under general anesthesia with double‐lumen endotracheal intubation and single‐lung ventilation. A 3.0 cm incision was made at the 5th intercostal space along the left midaxillary line. The thoracoscopic system with a 10 mm 30° rigid thoracoscope and conventional thoracic minimally invasive instruments (including curved graspers, ultrasonic scalpel, and endoscopic needle holders) was adopted for surgery. Thoracoscopic exploration identified a cystic‐solid mass with a diameter of ~20 mm along the phrenic nerve pathway adjacent to the left hilum, featuring medium‐soft consistency, well‐defined borders, and an intact capsule. The superior and inferior poles of the mass were densely adherent to the main phrenic nerve trunk, with no available dissectable interval, making intracapsular enucleation infeasible to avoid residual tumor tissue and irreversible nerve injury.

Intraoperatively, an ultrasonic scalpel was utilized to meticulously dissect surrounding tissues along the tumor capsule, followed by complete tumor resection and submission for intraoperative frozen section analysis. Frozen histopathology indicated a spindle‐cell tumor with no evident cellular atypia, consistent with schwannoma. Subsequently, the diseased segment of the phrenic nerve was transected using endoscopic scissors, resulting in a 25 mm nerve defect. Full‐thickness end‐to‐end suturing of the phrenic nerve was performed using a 5‐0 Prolene suture, with 3 stitches at the proximal stump and 4 stitches at the distal stump to ensure a tension‐free anastomosis. Post‐anastomosis, electrical stimulation of the distal anastomotic site via an electrocoagulation hook confirmed preserved phrenic nerve conductivity. A thoracic drainage tube was placed through the primary incision at the left 5th intercostal midaxillary line, and the thoracic cavity was closed after meticulous hemostasis. The total operative time was 69 min, with an estimated intraoperative blood loss of 10 mL.

Postoperative histopathological examination confirmed the diagnosis of benign schwannoma, and immunohistochemical staining showed positive expression of the S‐100 protein and SOX10, with a Ki‐67 proliferation index of <5%. No postoperative complications, including pulmonary infection, hemorrhage, or aggravated nerve injury, occurred. The thoracic drainage tube was removed on postoperative day 2, and the patient was discharged uneventfully on postoperative day 3, with a postoperative hospital stay of 3 days.

### 2.2. Case 2

A 22‐year‐old female patient was admitted to our hospital in February 2025 with a chief complaint of “incidentally detected right middle mediastinal mass for 4 months.” Four months prior, the patient was found to have a cystic density mass adjacent to the right mediastinum on chest CT during routine physical examination, measuring ~38 mm × 34 mm. The local hospital suspected a pericardial cyst and recommended regular radiographic follow‐up. One week before admission, repeated contrast‐enhanced chest CT revealed a heterogeneous low‐density mass adjacent to the right mediastinum, with well‐defined borders, dimensions of ~30 mm × 34 mm × 55 mm, a plain scan CT value of 34 HU, patchy and strip‐like enhancement post‐contrast, and mild compression of the adjacent superior vena cava and right atrium (Figure 2A,B). The patient was asymptomatic at admission, with no history of smoking, alcohol consumption, or familial malignant tumors. Physical examination showed midline trachea, no superficial lymphadenopathy, clear bilateral breath sounds without rales, and normal cardiac and abdominal findings, with an ECOG score of 0.

**Figure 2:**
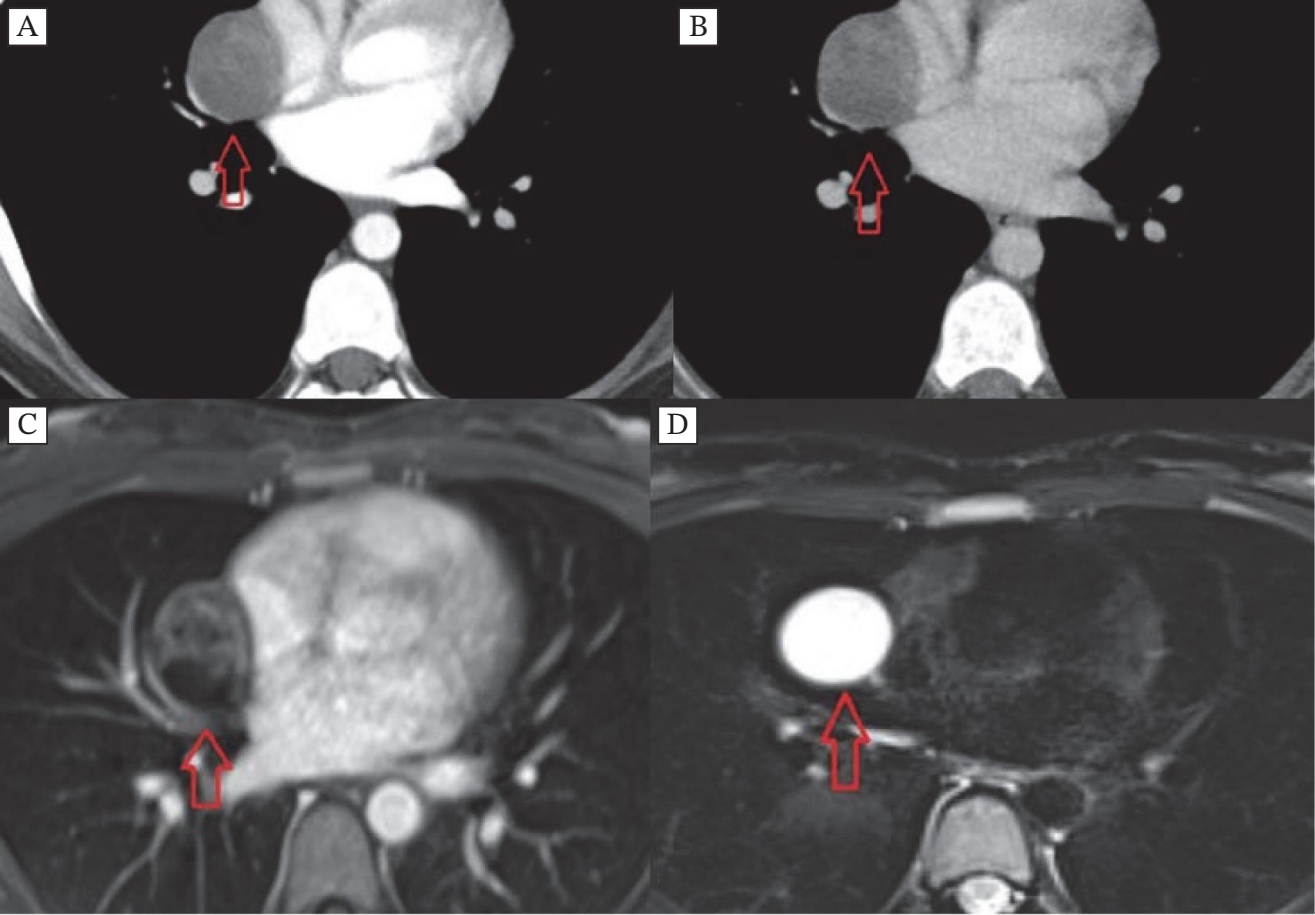
Chest imaging findings of Case 2. (A) Arterial phase of contrast‐enhanced chest CT showing heterogeneous enhanced mass in the right middle mediastinum (arrow indicates the tumor lesion); (B) venous phase of contrast‐enhanced chest CT showing continuous enhancement of the lesion and mild compression of peripheral vascular structures (arrow indicates the tumor); (C) contrast‐enhanced MRI T1WI showing slightly low signal of the mass (arrow indicates the lesion); (D) contrast‐enhanced MRI T2WI FS showing high heterogeneous signal of the tumor (arrow indicates the lesion).

After admission, contrast‐enhanced chest magnetic resonance imaging (MRI) was performed, demonstrating slightly low signal intensity on T1‐weighted imaging (T1WI), high signal intensity on T2‐weighted imaging fat saturation (T2WI FS), and slightly high signal intensity on diffusion‐weighted imaging (DWI). The lesion exhibited a heterogeneous internal signal, dimensions of ~32 mm × 27 mm × 51 mm, smooth borders, and no lobulation, with heterogeneous enhancement post‐contrast. These findings suggested a benign or borderline neoplasm, without excluding thymoma or neurogenic tumor (Figure 2C,D). Preoperative auxiliary examinations showed no abnormal indicators, and fine‐needle aspiration biopsy was not performed preoperatively.

The patient received uni‐VATS with double‐lumen endotracheal intubation and single‐lung ventilation in the left lateral decubitus position. A 3.0 cm incision was made at the 4th intercostal space of the right midaxillary line, with the same thoracoscopic system and surgical instruments as in Case 1. Thoracoscopic exploration revealed a cystic‐solid mass alongside the phrenic nerve in the right middle mediastinum, with medium consistency, intact capsule, and a clear dissection plane between the tumor and phrenic nerve perineurium, with no obvious adhesion.

Intracapsular enucleation was performed using an ultrasonic scalpel, with complete resection of the tumor tissue while strictly preserving the integrity of the main phrenic nerve trunk (Figure 3A,B). The specimen was placed in a retrieval bag and extracted via the surgical incision, followed by intraoperative frozen section analysis. Frozen histopathology confirmed a spindle‐cell tumor, consistent with schwannoma. A thoracic drainage tube was inserted through the primary incision at the right 4th intercostal midaxillary line, and the chest was closed after thorough hemostasis. The operation lasted 60 min, with an estimated intraoperative blood loss of 10 mL.

**Figure 3:**
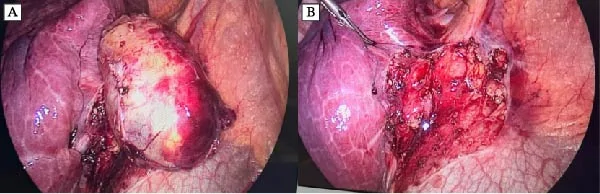
Intraoperative thoracoscopic views of tumor intracapsular enucleation in Case 2. (A) Pre‐enucleation intraoperative view showing the intact cystic‐solid phrenic nerve schwannoma adjacent to the right phrenic nerve; (B) post‐enucleation intraoperative view showing complete tumor resection and well‐preserved integrity of the phrenic nerve trunk without injury.

Postoperative histopathological examination verified the diagnosis of schwannoma, and immunohistochemical staining showed positive S‐100 protein and SOX10 expression, with a Ki‐67 proliferation index of 5% (Figure 4A,B). No perioperative complications were observed. The thoracic drainage tube was removed on postoperative day 2, and the patient was discharged smoothly on postoperative day 3, with a postoperative hospital stay of 3 days.

**Figure 4:**
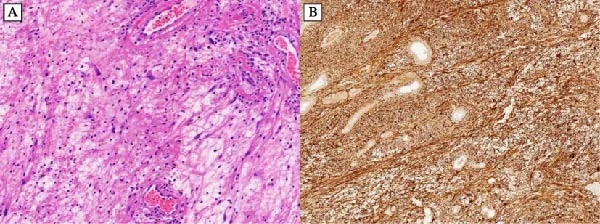
Pathological and immunohistochemical results of Case 2. (A) HE staining of tumor tissue showing spindle cell proliferation with no cellular atypia (×100 magnification); (B) immunohistochemical staining showing diffuse positive expression of S‐100 protein in tumor cells (×100 magnification).

### 2.3. Follow‐Up Results

Both cases received standardized regular follow‐up after discharge. Case 1 completed 12 months of follow‐up, and chest X‐ray at 3 months postoperatively demonstrated mild left diaphragmatic elevation (Figure 5A,B). Notably, the patient had no subjective symptoms of chest tightness or dyspnea, so no targeted intervention was administered. Chest CT at 12 months postoperatively showed stable diaphragmatic elevation without deterioration (Figure 5C), and pulmonary function tests revealed normal ventilatory function, indicating adaptive compensatory recovery of diaphragmatic function. Case 2 received 6 months of follow‐up, and chest X‐ray at 3 months postoperatively showed no tumor recurrence, diaphragmatic abnormality, or respiratory dysfunction, indicating satisfactory postoperative recovery.

**Figure 5:**
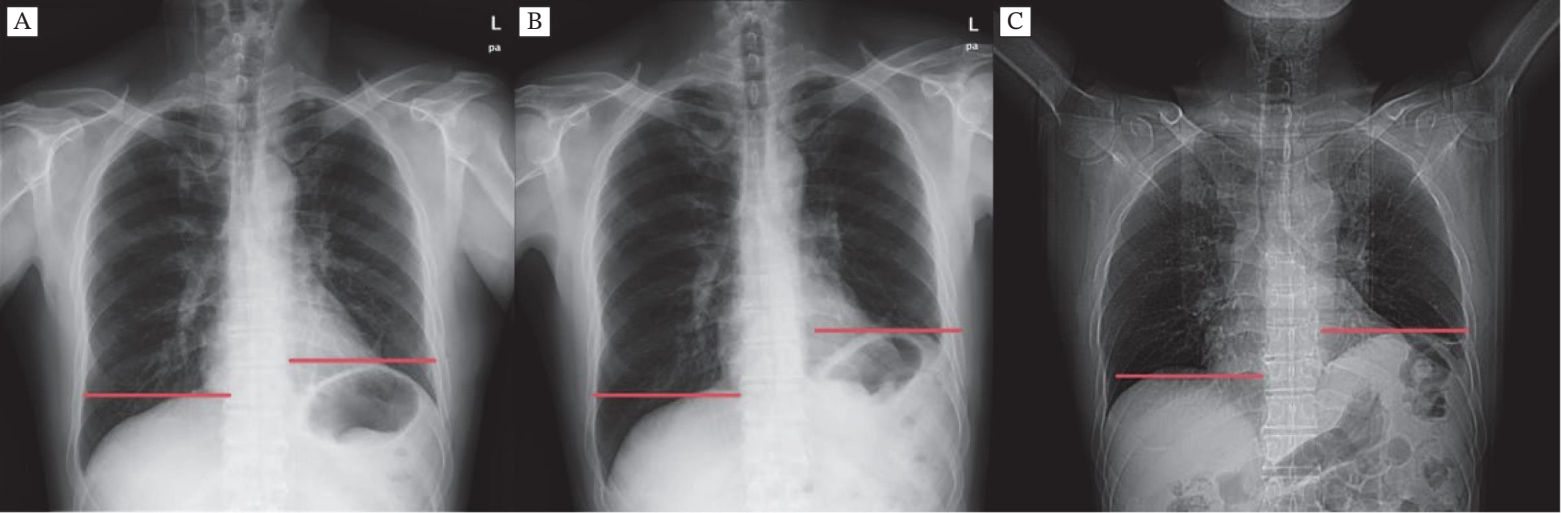
Postoperative follow‐up imaging findings of Case 1. (A) Chest radiograph on postoperative day 3 showing mild left diaphragmatic elevation (red lines mark the bilateral diaphragmatic contour); (B) chest radiograph at 3 months postoperatively showing stable left diaphragmatic elevation without progressive changes (red lines mark the bilateral diaphragmatic contour); (C) noncontrast chest CT at 12 months postoperatively confirming no tumor recurrence and stable diaphragmatic morphology.

## 3. Discussion

PNS is a rare benign neoplasm in clinical practice, with a peak incidence at 30–60 years of age and a male‐to‐female ratio of ~1:1.2 [3, 11]. The clinical manifestations of this tumor are closely correlated with its anatomical location: cervical PNS typically presents as a painless cervical mass, which may be accompanied by radiating shoulder pain with tumor enlargement [3, 12]; intrathoracic PNS is usually asymptomatic in the early stage due to the spacious thoracic cavity, and may manifest as chest tightness, chest pain, or dyspnea when the lesion enlarges and compresses adjacent organs and nerves [4, 6, 13]. The two cases in this study were both diagnosed with intrathoracic PNS, consisting of one male and one female aged 55 and 22 years, respectively, both incidentally detected during physical examination without clinical symptoms, which aligns with the early clinical features of intrathoracic PNS.

Imaging examination serves as the cornerstone of preoperative evaluation for PNS, with contrast‐enhanced chest CT and MRI as the first‐line diagnostic modalities [14, 15]. Characteristic contrast‐enhanced CT findings of PNS include round or fusiform masses along the nerve pathway with well‐defined borders, heterogeneous post‐contrast enhancement, cystic degeneration in partial cases, and an absence of calcification in most instances; typical MRI features include an isointense or slightly hypointense signal on T1WI, a hyperintense signal on T2WI, an occasional “target sign,” which aids in differential diagnosis from other neurogenic tumors [2]. It is noteworthy that imaging examinations can only preliminarily assess the tumor nature and location, failing to confirm phrenic nerve origin, leading to a low rate of accurate preoperative diagnosis. In the present two cases, preoperative contrast‐enhanced CT only suggested mediastinal neurogenic tumors, and the phrenic nerve origin was confirmed intraoperatively, consistent with previous literature reports [2, 14, 15].

Positron emission tomography‐CT (PET‐CT) has limited diagnostic value and low specificity in differentiating benign and malignant PNS and is only selectively indicated when malignant peripheral nerve sheath tumors or other malignant mediastinal neoplasms need to be excluded [6]. Fine‐needle aspiration biopsy carries a high false‐negative rate for PNS and entails a risk of iatrogenic nerve injury; thus, it is not recommended as a routine preoperative examination [12, 16]. In our two cases, preoperative multimodal imaging presented typical benign tumor features without any malignant suggestive signs, supporting the rationality of omitting PET‐CT and puncture biopsy to avoid unnecessary invasive trauma and medical resource waste. Definitive diagnosis of PNS relies on intraoperative findings and postoperative histopathological examination combined with immunohistochemical staining, among which positive S‐100 protein expression is a pivotal diagnostic marker [4, 11, 14].

Surgical resection is the gold‐standard treatment for PNS, with the core therapeutic principle of “complete tumor resection combined with maximal phrenic nerve function preservation” [3, 17, 18]. Intraoperative individualized surgical decision‐making is strictly based on the adhesive state and anatomical interface between the tumor and the phrenic nerve trunk. Intracapsular enucleation is suitable for patients with a clear dissection plane between the tumor and phrenic nerve perineurium without dense adhesion, which effectively maintains nerve continuity and markedly reduces the risk of postoperative diaphragmatic paralysis [3, 19]; in cases where tumors tightly adhere to or infiltrate the nerve parenchyma, intracapsular dissection cannot achieve radical resection, inevitably leading to residual tumor tissue and potential local recurrence. Under such circumstances, tumor resection combined with phrenic nerve reconstruction is mandatory to balance radical oncological resection and functional nerve protection [2]. In Case 1 of our study, intraoperative exploration confirmed inseparable dense adhesion between the tumor and main phrenic nerve trunk, rendering intracapsular enucleation technically unfeasible. After complete tumor resection, tension‐free end‐to‐end neurorrhaphy was performed to reconstruct nerve continuity. Postoperative follow‐up identified only asymptomatic mild diaphragmatic elevation, verifying the efficacy of this reconstructive strategy [20]. For nerve defects exceeding 3 cm after tumor resection, autologous intercostal nerve or sural nerve grafting is recommended to optimize postoperative functional recovery [21]. By contrast, Case 2 presented a clear tumor‐nerve anatomical interval, and intracapsular enucleation was successfully implemented to preserve intact phrenic nerve structure with uneventful functional recovery postoperatively.

Thoracoscopic surgery is the first‐choice surgical approach for intrathoracic PNS, while traditional thoracotomy is only reserved for cases with giant tumors or severe peritumoral adhesion that are unsuitable for thoracoscopic management [6, 18, 22]. Uni‐VATS has become a viable surgical option for appropriately sized intrathoracic PNS due to its minimal invasiveness, mild postoperative pain, rapid recovery, and satisfactory cosmetic outcomes. Pham et al. [23] reported a case of intrathoracic PNS treated with uni‐VATS, with the patient discharged uneventfully on postoperative day 4 and no tumor recurrence during follow‐up, validating the safety and efficacy of this technique. Both cases in this study underwent uni‐VATS with short operative time, minimal blood loss, brief hospital stay, and favorable surgical outcomes, further corroborating the clinical value of uni‐VATS for intrathoracic PNS.

Despite these advantages, uni‐VATS has definite technical limitations that must be acknowledged for objective clinical evaluation. The single‐port operating paradigm creates overlapping instrument angles and a steep learning curve for junior thoracic surgeons. Intraoperative misuse of ultrasonic energy devices may induce thermal or traction injury to the delicate phrenic nerve plexus. Moreover, this technique is not applicable for massively enlarged tumors or severely adhesive lesions that require extensive dissection [23]. Robotic thoracic surgery provides high‐definition three‐dimensional visualization and precise tremor‐filtered manipulation, yet its widespread application is restricted by excessive medical costs and limited clinical accessibility. In this study, we systematically supplemented practical technical details for uni‐VATS‐based PNS resection: a 10 mm 30° angled rigid thoracoscope was adopted for panoramic thoracic visualization, paired with curved fine‐tip graspers and precision ultrasonic scalpels for atraumatic dissection; and real‐time intraoperative electrical nerve stimulation was routinely performed to monitor phrenic nerve conductivity, effectively lowering the risk of iatrogenic nerve functional impairment.

Mediastinitis represents a severe and potentially lethal postoperative complication following intrathoracic surgical procedures. A recent scoping review by Cobzeanu et al. [24] systematically demonstrated that deep cervical and thoracic fascial space infections can rapidly propagate downward, leading to descending necrotizing mediastinitis, a condition characterized by aggressive inflammatory progression, high sepsis incidence, and an untreated mortality rate approaching 50%. For patients undergoing phrenic nerve resection and reconstructive anastomosis for mediastinal schwannoma, intraoperative subtle thoracic contamination, inadequate postoperative drainage, and occult soft tissue inflammation can all serve as triggering factors for mediastinal cellulitis and progressive necrotizing mediastinitis. Accordingly, standardized intraoperative protocols, including meticulous hemostasis, thorough thoracic cavity irrigation, and standardized postoperative drainage management combined with empirical anti‐infective prophylaxis, are indispensable to mitigate this life‐threatening surgical complication.

According to the World Health Organization (WHO) tumor classification criteria, PNS is classified as a grade I benign neoplasm, characterized by slow growth and excellent long‐term prognosis. Following complete surgical resection, the 10‐year disease‐free survival rate exceeds 95%, with an extremely low recurrence rate [6, 25]. Nevertheless, the follow‐up duration in the present study (12 months for Case 1 and 6 months for Case 2) remains relatively short for this slowly recurring tumor, and prolonged longitudinal follow‐up is essential to monitor potential late recurrence. Additionally, this study only adopted qualitative assessment of postoperative diaphragmatic and respiratory function, lacking objective quantitative indicators such as dynamic diaphragmatic motion ultrasonography and graded pulmonary function testing, which constitutes a major limitation of this clinical report. Clinically, postoperative unilateral diaphragmatic elevation may reduce exercise pulmonary tolerance by 20%–30% in elderly individuals or patients with preexisting pulmonary comorbidities; however, young and physically healthy patients can generally achieve satisfactory adaptive functional compensation without obvious clinical symptoms [21, 26, 27].

Collectively, intrathoracic PNS is a rare benign mediastinal lesion with a challenging preoperative differential diagnosis. Uni‐VATS serves as a feasible, safe, and minimally invasive surgical option for appropriately sized intrathoracic PNS lesions. Individualized surgical strategies, tailored according to intraoperative tumor‐phrenic nerve anatomical relationships (either intracapsular enucleation or nerve reconstruction), enable maximal preservation of phrenic nerve function and favorable short‐term postoperative outcomes. Further long‐term follow‐up studies and multicenter controlled trials are warranted to validate the long‐term therapeutic efficacy of uni‐VATS and establish standardized surgical management guidelines for rare intrathoracic PNS.

## Author Contributions

Conceptualization, formal analysis, writing – original draft: Junjun Ni. Methodology, resources: Junjun Ni and Minglei Yang. Supervision, writing – review and editing: Minglei Yang. Data curation, literature review: Yangbo Qiu and Rui Li.

## Funding

This work was supported by the Key Joint Project of Basic Public Welfare Research in Zhejiang Province (Grant LHDMD23H160001), the General Scientific Research Project of Zhejiang Provincial Department of Education (Grant Y202352628), and the Major Tackling Project of High‐end Medical and Health Team in Ningbo City (Grant 2022030208).

## Ethics Statement

This case report has been prepared in accordance with the 2013 CARE Checklist.

## Consent

Written informed consent was obtained from the patient for the publication of this case report and accompanying images. A copy of the written consent is available for review by the Editor‐in‐Chief of this journal upon request.

## Conflicts of Interest

The authors declare no conflicts of interest.

## Data Availability

The data that support the findings of this study are available from the corresponding author upon reasonable request.
